# Radical surgery versus standard surgery for primary cytoreduction of bulky stage IIIC and IV ovarian cancer: an observational study

**DOI:** 10.1186/s12885-015-1525-1

**Published:** 2015-08-13

**Authors:** Yulan Ren, Rong Jiang, Sheng Yin, Chao You, Dongli Liu, Xi Cheng, Jie Tang, Rongyu Zang

**Affiliations:** 1 Department of Gynecologic Oncology, Fudan University Shanghai Cancer Center, Shanghai 200032, China; 2 Department of Radiology, Fudan University Shanghai Cancer Center, Shanghai 200032, China; 3 Division of Gynecology Oncology, Department of Obstetrics and Gynecology, Zhongshan Hospital, Fudan University, Shanghai 200032, China

**Keywords:** Radical surgery, Extensive upper abdominal surgery, Ovarian cancer, Upper abdominal disease, Survival

## Abstract

**Background:**

The aim of this study was to evaluate the survival benefit of radical surgery with additional extensive upper abdominal procedures (EUAS) for the treatment of stage IIIC and IV ovarian cancer with bulky upper abdominal disease (UAD).

**Methods:**

An observational study was conducted between 2009 and 2012 involving two different surgical teams. Team A was composed of the “believers” in EUAS and Team B the “non-believers” in EUAS. Patients were divided into a radical surgery group (EUAS group) or a standard surgery group (non-EUAS group) according to whether or not they had received EUAS. All patients underwent primary cytoreductive surgery with the goal of optimal debulking (≤1 cm); this was reviewed in the pelvis, middle abdomen, and upper abdomen. The baseline for the two groups was optimal cytoreduction in both the pelvis and middle abdomen. Progression-free survival (PFS) was evaluated.

**Results:**

Radical surgery was performed in 70.7 % (82/116) and 12.7 % (30/237) of the patients by Teams A and B, respectively. The study groups had similar clinicopathologic characteristics. The median PFS and OS were significantly improved in the radical surgery group, compared with standard surgery groups (PFS: 19.5 vs. 13.3 months, HR: 0.61; 95 % CI: 0.46–0.80, *P* < 0.001; OS: not reached vs. 39.3 months, HR: 0.47; 95 % CI: 0.30–0.72, *P* < 0.001). Positive predictors of complete cytoreduction were treatment with neoadjuvant chemotherapy, improved American Society of Anesthesiologists performance status, and the absence of bowel mesenteric carcinomatosis.

**Conclusions:**

Radical surgery lengthens the PFS and overall survival times of ovarian cancer patients with bulky UAD. However, a well-designed randomized trial is needed to confirm the present results.

## Background

Epithelial ovarian cancer (EOC) is the most lethal of all gynecological cancers [1]. The goal of primary cytoreduction for advanced EOC is advocated to be no visible residual disease,which has been confirmed in several studies, but only less than 30 % of women with bulky upper abdominal disease (UAD) can achieve complete cytoreduction [2, 3]. Thus, it still remains controversial as to whether or not patients with bulky UAD can benefit from upper abdominal procedures (EUAS). It has been suggested that upper abdominal procedures should only be performed when complete or optimal cytoreduction is attainable [4–6].

In China, only a few surgeons are willing to undertake EUAS because most lack the relevant surgical skills, or there is tension between patients and physicians regarding the invasiveness of the treatment. Consequently, to date, there have been no Chinese studies in this area [7]. Most of the surgeons tend to accept neoadjuvant chemotherapy followed by surgery as the standard approach, which is in line with the result of EORTC 55971 study reported in 2010 [8].

Herein, we define *radical surgery* as the EUAS procedures complementing an optimal cytoreduction within the middle abdomen and the pelvis. These procedures include diaphragmatic peritonectomy, hepatic resection, splenectomy, distal pancreatectomy, cholecystectomy, and the resection of tumor on the surface of upper abdominal organs. *Standard surgery* is defined as the optimal surgical outcome achieved in both the middle abdomen and the pelvis (including small and/or large bowel resections), and the subsequent attempt to resect tumor nodes measuring ≥1 cm in the upper abdomen. An exploratory study was conducted to compare the survival after radical surgery with standard surgery in patients with bulky stage IIIC and IV ovarian cancer.

## Methods

### Patients

A single institute observational study was conducted between 2009 and 2012. Patients who were diagnosed with stage IIIC or IV epithelial ovarian cancer with bulky UAD were identified and the data were collected retrospectively, which was approved by Fudan University Shanghai Cancer Center Institutional Review Board (SCCIRB-090371-2). The residual disease was evaluated in the pelvis, middle abdomen, and upper abdomen, respectively. In the standard surgery (non-EUAS surgery) group, patients with residual disease measuring ˃1 cm in the pelvis and middle abdomen were excluded.

Medical records were abstracted for the following data: age at primary cytoreduction; International Federation of Gynecology and Obstetrics (FIGO) stage; histology; tumor grade; Eastern Cooperative Oncology Group (ECOG) performance status; American Society of Anesthesiologists (ASA) score; preoperative serum cancer antigen (CA125); ascites and extent of disease (categorized as solitary, localized, and carcinomatosis defined as diffused involving of the peritoneal surfaces) at primary surgery; cytoreductive procedures; residual disease after primary cytoreduction; surgeons involved; type of front-line chemotherapy; estimated blood loss; intraoperative blood transfusion; operative time; intensive care unit (ICU) stay; length of hospitalization; postoperative 30-day morbidity and mortality; progression-free survival (PFS), follow-up time; and survival time. The computed tomography (CT) or magnetic resonance imaging (MRI) scan was reviewed by the radiologist (CY) in all possible patients to re-evaluate the upper abdominal disease.

### Definitions

There were two different surgical teams: Team A was the “believers” and “deeds over words” regarding EUAS procedures, who attempted to resect any macroscopic disease; Team B was “non-believers” in EUAS.

PFS was defined as the time from initial treatment to the diagnosis of the first recurrence or last follow-up, whichever came first. Overall survival (OS) was defined as the time from initial treatment to death or last follow-up. Recurrence was diagnosed by one or more of the following: physical examination; elevated CA-125 levels as defined by the Gynecologic Oncology Intergroup [9]; and radiological imaging.

The abdominal tumor site (pelvis, middle abdominal, and upper abdominal disease) at primary cytoreduction was defined as previously described [10]. Optimal cytoreduction was defined as residual disease measuring ≤1 cm, but the cut-off points of 0 cm and 0.5 cm were also used to evaluate the impact on survival.

### Statistical analysis

Statistical analysis was performed using the SPSS software package for Windows (version 16.0). The Chi-square or Mann–Whitney U tests were used to identify differences in the baseline level between the two groups. Median survival was evaluated using the Kaplan–Meier method and differences were determined using the log-rank test. The Cox proportional hazards regression model was used to identify prognostic factors. Logistic regression analysis was conducted to detect the predictors of complete cytoreduction. A *P*-value of <0.05 was considered as being statistically significant.

## Results

### Baseline and patient characteristics

Three hundred and fifty-three patients were included in this observational study. Of these patients, 112 received radical surgery including EUAS procedures, and 241 received standard surgery. In radical surgery group, Team A did 82 cases (73.2 %) radical surgery; and Team B did 30 (26.8 %) radical surgery. Altogether Team A did surgery in 116 patients, and Team B did surgery in 237 patients. Patient baseline characteristics as well as the imaging findings regarding the disease within the upper abdomen were similar between the two groups (Tables 1 and 2). In the radical surgery group, eight (7.1 %) patients were upstaged for pleural metastasis and 21 (18.8 %) were diagnosed with stage IV ovarian cancer in the EUAS group as compared with 26 (10.8 %) patients in the non-EUAS group. In the radical surgery group, the optimal cytoreduction achieved in the pelvis and middle abdomen was 98.2 % and 97.3 %, respectively; all patients in the standard surgery group underwent optimal cytoreduction. However, more patients in the radical surgery group had microscopic residual disease, both in the pelvis (82.1 % versus 53.5 %) and the middle abdomen (53.6 % versus 28.6 %) when compared with those patients in the control group.

**Table 1:** Baseline of patient characteristics

Characteristic	Radical surgery group	Standard surgery group	*P* value^*^
Median age (range)	56 years (35–82)	56 years (26–79)	1.000
FIGO stage			0.045
Stage IIIC	91(81.2 %)	215(89.2 %)	
Stage IV	21(18.8 %)^b^	26(10.8 %)	
Primary tumor			0.535
Epithelial ovarian cancer	111 (99.1 %)	240 (99.6 %)	
Fallopian tube cancer	1 (0.9 %)	0 (0 %)	
Primary peritoneal cancer	0 (0 %)	1 (0.4 %)	
Histology			0.084
Serous	100(89.3 %)	202(83.8 %)	
Mucinous	0(0 %)	2(0.8 %)	
Endometrioid	2(1.8 %)	2(0.8 %)	
Clear cell	2(1.8 %)	5(2.1 %)	
Others	8(7.2 %)	30(12.4 %)	
Grade			0.787
Grade1	0(0 %)	2(0.8 %)	
Grade 2	9(8.0 %)	16(6.6 %)	
Grade 3	102(91.1 %)	218(90.5 %)	
NA	1(0.9 %)	5(2.1 %)	
ECOG performance status			0.116
0	57(50.9 %)	95(39.4 %)	
1	49(43.8 %)	126(52.3 %)	
2	6(5.4 %)	20(8.3 %)	
ASA status			0.358
1	59(52.7 %)	107(44.4 %)	
2	51(45.5 %)	127(52.7 %)	
3	2(1.8 %)	7(2.9 %)	
Preoperative serum CA125			0.245
Median serum level (range)	1320 U/ml (67.2–77050)	1725 U/ml (32.1–39145)	
Neoadjuvant chemotherapy			0.421
Yes	20 (17.9 %)	35 (14.5 %)	
No	92 (82.1 %)	206 (85.5 %)	
Ascites			0.472
Median volume (range)	1350 ml (0–7000)	1500 ml (0–10000)	
Bowel mesenteric carcinomatosis			0.544
Yes	78 (69.6 %)	160 (66.4 %)	
No	34 (30.4 %)	81 (33.6 %)	
Residual disease in pelvis			<0.001
0 cm	92(82.1 %)	129(53.5 %)	
0.1–0.5 cm	15(13.4 %)	71(29.5 %)	
0.5–1 cm	3(2.7 %)	41(17.0 %)	
>1 cm	2(1.8 %)	0(0 %)	
Residual disease in middle abdomen			<0.001
0 cm	60(53.6 %)	69(28.6 %)	
0.1–0.5 cm	40(35.7 %)	93(38.6 %)	
0.5–1 cm	9(8.0 %)	79(32.8 %)	
>1 cm	3(2.7 %)	0(0 %)	
Total	112	241	

Abbreviations: *FIGO* International Federation of Gynecology and Obstetrics, *ECOG* Eastern Cooperative Oncology Group, *ASA* American Society of Anesthesiologists, *NA* not available

^*^Tested by Chi-square or Mann–Whitney U. ^b^ Thoracic exploration was performed in 13 patients and 8 patients were upstaged for pleural metastasis

**Table 2:** Preoperative imaging for the evaluation of upper abdominal disease

Tumor site	Radical surgery group	Standard surgery group	*P* value^*^
Right diaphragm	29 (76.3 %)	49 (68.1 %)	0.364
Left diaphragm	6 (15.8 %)	19 (26.4 %)	0.207
The surface of liver	9 (23.7 %)	12 (16.7 %)	0.373
The surface of spleen	0 (0 %)	3 (4.2 %)	0.202
Portahepatis	7 (18.4 %)	6 (8.3 %)	0.119
Perisplenicregion	9 (23.7 %)	17 (23.6 %)	0.993
Spleen parenchyma	4 (10.5 %)	2 (2.8 %)	0.099
Lesser omentum	10 (26.3 %)	10 (13.9 %)	0.108
Diaphragmatic lymph node	24 (64.9 %)	35 (48.6 %)	0.107
Total	38	72	

^*^Tested by Chi-square

### Surgical outcomes

There were significant differences between radical surgery involving EUAS and standard surgery in terms of estimated blood loss, intraoperative blood transfusion, operative time, ICU stay, and length of hospitalization (Table 3). In the EUAS group, optimal cytoreduction was performed in 107 patients (95.5 %), and in 76 patients (67.9 %) complete cytoreduction was achieved in the upper abdomen. However, no patients achieved complete cytoreduction in the control arm, and only 43.6 % received optimal surgery.

**Table 3:** Surgical outcomes between radical surgery with extensive upper abdominal procedures and standard surgery

Variable	Radical surgery group (n = 112)	Standard surgery group (n = 241)	*P* value^*^
Residual disease, in overall			<0.001
0 cm	46 (41.1 %)	0 (0 %)	
0.1–0.5 cm	46 (41.1 %)	27 (11.2 %)	
0.5–1 cm	15 (13.4 %)	78 (32.4 %)	
>1 cm	5 (4.5 %)	136 (56.4 %)	
Residual disease in upper abdomen			<0.001
0 cm	76(67.9 %)	0(0 %)	
0.1–0.5 cm	24(21.4 %)	30(12.4 %)	
0.5–1 cm	10(8.9 %)	75(31.1 %)	
>1 cm	2(1.8 %)	136(56.4 %)	
Estimated blood loss			<0.001
Median volume	800 ml	600 ml	
(range)	(100–4000)	(100–3000)	
Intraoperative blood transfusion			<0.001
Median volume	800 ml	400 ml	
(range)	(0–3200)	(0–2400)	
Operative time			<0.001
Median	171 min	124 min	
(range)	(75–360)	(51–318)	
ICU stay			0.018
Yes	28 (25.0 %)	35 (14.5 %)	
No	84 (75.0 %)	206 (85.5 %)	
Length of hospitalization			<0.001
Mean	19.96 days	10.39 days	
(range)	(4–190)^b^	(4–42)	

^*^Tested by Chi-square or Mann–Whitney U.^b^33 patients (29.5 %) in radical surgery group participated in a phase II clinical trial on intraperitoneal chemotherapy and were assigned to receive IP chemotherapy for 4 cycles weekly post operative in hospital, whereas only 7 patients (1.7 %) in standard surgery group participated in this trial and received IP chemotherapy. One patient stayed in hospital for 190 days because she received all the cycles of IP and IV chemotherapy in hospital

The extensive upper abdominal procedures performed in the radical surgery group included diaphragm peritonectomy, full-thickness diaphragm resection, resection of the lesser omentum, splenectomy, liver resection, distal pancreatectomy, cholecystectomy, thoracic exploration, and resection of tumor on the surface of the liver, stomach, spleen, the gallbladder fossa and the renal capsule (Table 4). In the standard surgery group, only a few procedures involving upper abdominal surgery were conducted (Table 5). Radical surgery was performed in 73.2 % (82/112) and 12.4 % (30/241) of the patients by Teams A and B, respectively. When the data were divided into two periods from June 2009 to June 2011 and from July 2011 to December 2012, the level of performance of EUAS was elevated in the case of both teams (Table 6). The positive predictors of complete cytoreduction were as follows: undergoing neoadjuvant chemotherapy; better ASA performance status; and the absence of bowel mesenteric carcinomatosis (*P* = 0.006, *P* = 0.014 and *P* = 0.026, respectively; Table 7).

**Table 4:** The procedures of extensive upper abdominal surgery in radical surgery group (n = 112)

Procedure	No. of patients	Percent
Left side diaphragm peritonectomy	1	0.9 %
Right side diaphragm peritonectomy	79	70.5 %
Both sides diaphragm peritonectomy	20	17.9 %
Full-thickness diaphragm resection	7	6.3 %
Thoracic exploration	13	11.6 %
Resection of lesser omentum	35	31.3 %
Splenectomy	17	15.2 %
Liver resection	8	7.1 %
Distal pancreatectomy	2	1.8 %
Cholecystectomy	1	0.9 %
Resection of the tumor on the surface of liver	27	24.1 %
Resection of the tumor on the surface of stomach	8	7.1 %
Resection of the tumor on the surface of spleen	5	4.5 %
Resection of the tumor in the gallbladder fossa	5	4.5 %
Resection of the renal capsule	1	0.9 %

**Table 5:** The procedures of upper abdominal surgery in standard surgery group (n = 241)

Procedure	No. of patients	Percent
Diaphragm stripping	4	1.7 %
Diaphragm peritonectomy	0	0 %
Full-thickness diaphragm resection	0	0 %
Thoracic exploration	0	0 %
Resection of lesser omentum	6	2.5 %
Splenectomy	8	3.3 %
Liver resection	0	0 %
Distal pancreatectomy	1^a^	0 %
Cholecystectomy	0	0 %
Resection of the tumor on the surface of liver	3	1.2 %
Resection of the tumor on the surface of stomach	2	0.8 %
Resection of the tumor on the surface of spleen	1	0.4 %
Resection of the tumor in the gallbladder fossa	0	0 %
Resection of the renal capsule	0	0 %

^a^Distal pancreatectomy was mentioned in this patient’s surgical records, however pathology report showed there was no pancreatic tissue in the pathological sections

**Table 6:** Patients’ distributions of extensive upper abdominal surgery by surgeons

	Years of the data			
Groups	2009.6–2011.6		2011.7–2012.12	
	Team A	Team B	Team A	Team B
Radical surgery group (n = 112)	29 (65.9 %)	9 (8.8 %)	53 (73.6 %)	21 (15.6 %)
Standard surgery group (n = 241)	15 (34.1 %)	93 (91.2 %)	19 (26.4 %)	114 (84.4 %)
Total	44	102	72	135

Team A described as the “believers” and “deed over words” in EUAS (extensive upper abdominal surgery) procedures, trying to resect any macroscopic disease, and did 82 cases (73.2 %) radical surgery; Team B not believing EUAS, and only did 30 (26.8 %) radical surgery

**Table 7:** Variables affecting primary surgical outcomes (R0, R0.5) in patients with bulky stage IIIc and IV ovarian cancer^a,b^

Variables	B	S.E,	*P* value	RR	95 % C.I.	
					Upper	Lower
**R0**						
Neoadjuvant chemotherapy	−1.004	0.367	0.006	0.366	0.178	0.752
ASA score	0.772	0.314	0.014	2.165	1.170	4.006
Bowel mesenteric carcinomatosis^c^	0.718	0.323	0.026	2.051	1.008	3.866
**R0.5**						
Neoadjuvant chemotherapy	−0.907	0.301	0.003	0.404	0.224	0.729
ECOG performance	0.639	0.190	0.001	1.895	1.307	2.747

*B* beta coefficient, *SE* standard error, *RR* relative risk, *95* % *CI* 95 % confidence interval, *R0* complete cytoreduction, *R0.5* cut-off point of residual disease in overall all was 0.5 cm, *ASA* American Society of Anesthesiologists, *ECOG* Eastern Cooperative Oncology Group

^a^Logistic regression analysis

^b^ Variable “surgical team” was the most significant determinant of surgical outcomes, but it was not included into this model

^c^ It is a variable found in surgery, but interestingly, it is a predictor of complete cytoreduction not the predictor of small residual disease of 0.5 cm, so it was included in the Logistic regression model

In the standard surgery group, one patient died at 30 days after surgery because of a bowel obstruction, abdominal hemorrhage, and disease progression. However, there was no surgery-related death in the radical surgery group. The rate of Memorial Sloan-Kettering Cancer Centre (MSKCC) Grade III/IV complications in the two groups were 8.0 % (9/112) and 2.9 % (7/241), respectively. Thoracentesisor chest tube placement was more common in the radical surgery group (8.0 % versus 1.7 %; Table 8). There was no significant difference in morbidity and mortality between Teams A and B (Table 9).

**Table 8:** Morbidity and mortality between EUAS and non-EUAS groups

Complications	EUAS group (n = 112)	Non- EUAS group (n = 241)
Thoracentesis/chest tube placement	9(8.0 %)	4(1.7 %)
Pulmonaryembolism	1(0.9 %)	1(0.4 %)
Deep venous thrombosis	1(0.9 %)	0
Cerebral infarction	0	1(0.4 %)
Bowel obstruction	2(1.8 %)	8(3.3 %)
Wound infection	2(1.8 %)	2(0.8 %)
Abdominalinfections	1(0.9 %)	13(5.4 %)
Pneumonia	1(0.9 %)	1(0.4 %)
Urinary tract infection	0	1(0.4 %)
Gastroenteritis	1(0.9 %)	0
Heart failure/arrhythmia	1(0.9 %)	0
Relaparotomy for hemorrhage	1(0.9 %)	1 (0.4 %)
Blood transfusion for hemorrhage	0	2(0.8 %)
Median PRBC	0	8u/4u^a^
Intestinal fistula	0	1(0.4 %)
MSKCC Grade III/IV^b^	9 (8.0 %)	7 (2.9 %)
Mortality (MSKCC Grade V)	0	1(0.4 %)

EUAS: extensive upper abdominal surgery. PRBC: Packed Red Blood Cell

^a^Two patients required blood transfusion for hemorrhage, with one received 8u PRBC and the other one received 4u PRBC. ^b^Memorial Sloan-Kettering Cancer Center (MSKCC) surgical secondary events gradingsystem, see reference

**Table 9:** Morbidity and mortality by surgeons

Complications	Team A (n = 116)	Team B (n = 237)
Thoracentesis/chest tube placement	6(5.2 %)	7(3.0 %)
Pulmonaryembolism	1(0.9 %)	1(0.4 %)
Deep venous thrombosis	0	1(0.4 %)
Cerebral infarction	1(0.9 %)	0
Bowel obstruction	0(0.9 %)	10(4.2 %)
Wound infection	1(0.9 %)	3(1.3 %)
Abdominal infections	4(3.4 %)	10(4.2 %)
Pneumonia	0	2(0.8 %)
Urinary tract infection	0	1(0.4 %)
Gastroenteritis	1(0.9 %)	0
Heart failure/arrhythmia	1(0.9 %)	0
Relaparotomy for hemorrhage	0(0.0 %)	2 (0.8 %)
Blood transfusion for hemorrhage	0	2(0.8 %)
Intestinal fistula	0	1(0.4 %)
MSKCC Grade III/IV	7 (6.0 %)	9 (3.8 %)
Mortality (MSKCC Grade V)	0	1(0.4 %)

Team A did 82 cases (73.2 %) radical surgery; and Team B only did 30 (26.8 %) radical surgery

There were 2 cases of relaparotomy for hemorrhage, 2 blood transfusion for hemorrhage, 1 intestinal fistula and 1 death in Team B, with no case in Team A. Since there were more radical surgery in Team A, 6 cases (5.2 %) had chest tube placement in Team A, compared with 7 (3.0 %) in Team B

### Survival predictors

**Fig. 1:**
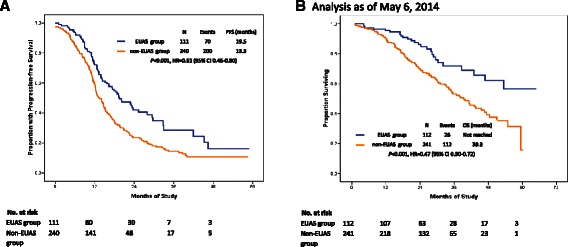
Survival analysis of EUAS group vs. non-EUAS group in bulky stage IIIC and IV EOC. **a**: Progression-free survival curve; **b**: Overall survival curve (Analysis as of May 6, 2014)

The median follow-up time was 25.3 (range, 16–65) months. The median PFS was 19.5 months and 13.3 months in the radical surgery and standard surgery groups, respectively (*P* < 0.001; hazard ratio (HR), 0.61; 95 % confidence interval (CI), 0.46–0.80; Fig. 1a), with an estimated 5-year PFS of 15 % and 10 %, respectively. The median survival time was 39.3 months in the standard surgery group; however, in the radical surgery group the median survival time was not reached (*P* < 0.001; HR, 0.47; 95 % CI, 0.30–0.72) (Fig. 1b). Residual disease in the upper abdomen (HR, 1.29; 95 % CI, 1.16–1.44; *P* = 0.002) and FIGO stage (HR, 1.62; 95 % CI, 1.16–2.27; *P* = 0.001) were found to be the predictors of PFS using Cox regression analysis (Table 10).

**Table 10:** Univariate and multivariate analysis of progression-free survival

Characteristic	N	Median PFS	Univariate	Multivariate	
		(months)	*P* value	HR (95 %CI)	*P* value
FIGO stage			0.004	1.62 (1.16–2.27)	0.001
IIIC	305	15.3			
IV	46	11.4			
Residual disease in upper abdomen			<0.001	1.29 (1.16–1.44)	0.002
0 cm	75	23.3			
0–0.5 cm	54	17.8			
0.5–1 cm	85	13.5			
>1 cm	137	12.6			
Total	351	14.7			

Abbreviations: *N* number of patients, *FIGO* International Federation of Gynecology and Obstetrics, *PFS* progression-free survival, *HR* hazard ratio, *95* % *CI* 95 % confidence interval

**Fig. 2:**
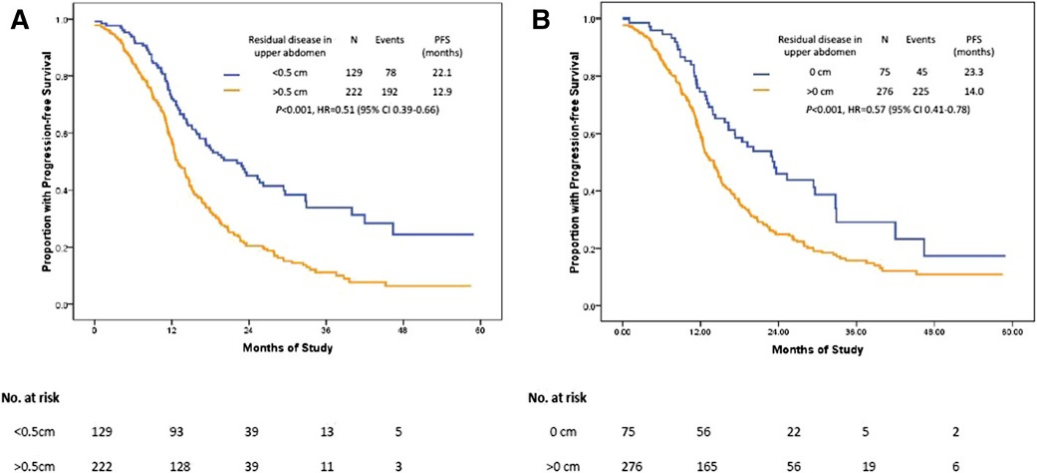
PFS by residual disease in the upper abdomen after primary cytoreductive surgery. **a**: comparison of residual disease between <0.5 cm vs. >0.5 cm; **b**: comparison of residual disease between 0 cm vs. >0 cm

**Fig. 3:**
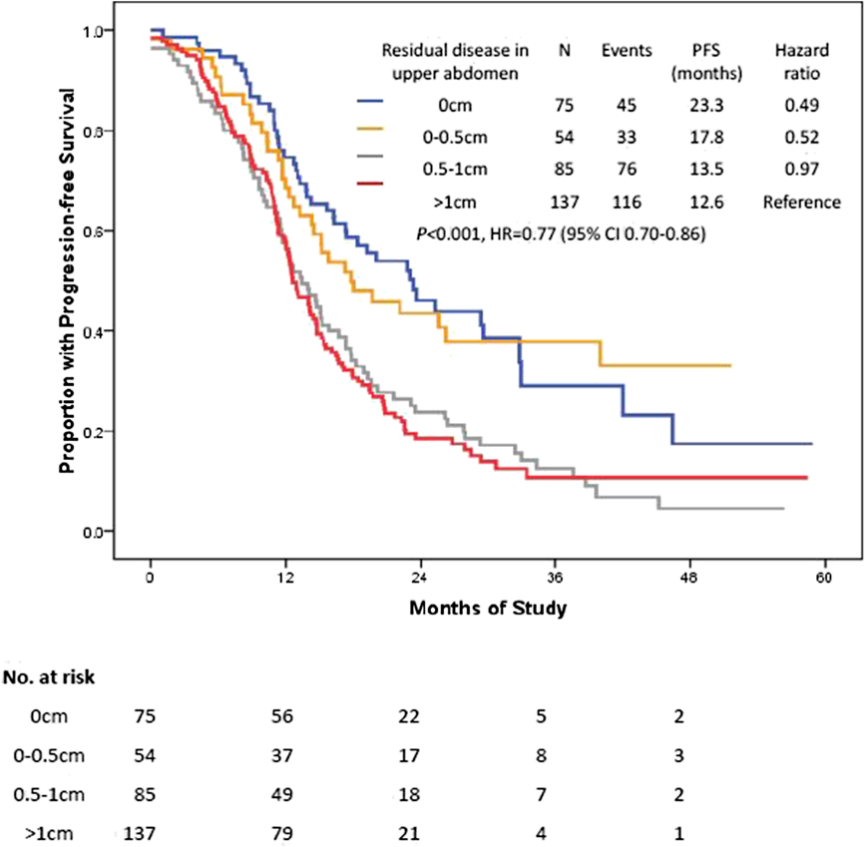
PFS by residual disease in upper abdomen after primary cytoreductive surgery. A comparison of residual disease among 0 cm, 0–0.5 cm, 0.5–1 cm, >1 cm

Patients with residual disease in the upper abdomen measuring <0.5 cm had a median PFS of 22.1 months, as compared with 12.9 months in patients with residual disease measuring >0.5 cm (*P* < 0.001; HR, 0.51; 95 % CI, 0.39–0.66; Fig. 2a). Moreover, the median PFS could reach 23.3 months in patients with microscopic disease in the upper abdomen (Figs. 2b and 3).

## Discussion

### The role of radical surgery with an additional upper abdominal cytoreduction

It is well established that EUAS could increase the proportion of patients achieving optimal cytoreduction as well as complete cytoreduction [4, 5, 11–13]. However, it is still unclear as to which patients would benefit from EUAS. Rodriguez and colleagues reviewed 2655 patients enrolled in the Gynecologic Oncology Group 182 trial from 2001 to 2004 who had achieved optimal cytoreduction (<1 cm) [4]. A total of 482 (18.1 %) patients received upper abdominal procedures. These authors reported that both the PFS (18.2 versus 14.8 months; *P* < 0.01) and OS (49.8 versus 43.7 months; *P* = 0.01) were higher in patients who did not require an upper abdominal procedure relative to patients who required upper abdominal procedures; they suggested that requiring upper abdominal surgery was an indicator of more extensive disease, which would have a negative impact on survival. When subgroup analysis was performed in patients with a high disease burden in the upper abdomen (n = 1636), it did not reveal an improved PFS (*P* = 0.43) in the 482 (29 %) patients who had undergone upper abdominal surgery relative to patients who had not, while the OS exhibited a modest improvement of 3.8 months. However, in the subset of patients who had received upper abdominal surgery, when considering postoperative residual disease, both the PFS (20.2 versus 13.7 months; *P* < 0.01) and the OS (54.6 versus 40.4 months; *P* < 0.01) were significantly prolonged in patients with complete cytoreduction (n = 141) relative to patients with minimal residual disease measuring <1 cm (n = 341). The authors concluded that in patients who required upper abdominal surgery, only the increased rate of complete cytoreduction could translate into prolonged OS [4]. However, regarding the MSKCC studies, their data demonstrated that EUAS significantly improved PFS and OS [13, 14]. In addition, the most recent study from this center has shown that patients with macroscopic residual disease ≤1 cm who required EUAS did not have a worse OS than those who did not require EUAS (45 months versus 52 months; *P* = 0.56) [5].

Unlike previous studies [4–6, 11–14], the current study was unique with respect to patient enrollment. First, it was an observational study and we only included patients from a single institution with bulky upper abdominal disease, who had achieved optimal primary resection in both the pelvis and middle abdomen in a recent period. Second, although patient selection bias still existed in the present study, the difference in the inherent tumor biology between the radical surgery group and the standard surgery group was balanced to a certain degree. Consequently, we could specifically analyze the role of EUAS and obtain results as to whether or not patients could obtain a survival benefit from EUAS.

In the present study, a 6.2-month prolongation in the median PFS was observed in patients who had undergone additional EUAS. Although the data were immature, a significant difference in OS was also observed. Because of the EUAS procedures, eight patients were upstaged for pleural metastasis. Therefore, thoracic exploration in EUAS could obtain an exact FIGO staging in patients with a heavy diaphragm tumor burden, especially those with an untapped pleural effusion.

As diaphragmatic peritonectomy was performed most frequently regarding EUAS, pleural effusion was the most common morbidity [15]. However, symptomatic pleural effusion can be well managed through thoracentesis or chest tube placement. The other morbidities such as pulmonary embolism, deep venous thrombosis, bowel obstruction, abdominal infections, and wound infection were not significantly increased in the radical surgery group. In addition, there was no surgery-related death in the radical surgery group. Although EUAS procedures were complicated, radical surgery did not increase the morbidity and mortality when compared with standard surgery.

### “Maximal” cytoreduction and residual disease in bulky ovarian cancer

Upper abdominal surgical procedures have proved to be more practical than most surgeons had anticipated. In our cohort, 89.3 % of the patients in the radical surgery group achieved residual disease measuring <0.5 cm and showed a survival advantage (Table 3). Additionally, the surgical outcome was accurately predicted by logistic regression analysis of CT/MRI scan data regarding the upper abdomen (Table 11). Moreover, residual disease in the upper abdomen was found to be one of the predictors of PFS (Table 10).

**Table 11:** Prediction of complete cytoreduction by diaphagmatic imaging findings^*^

Tumor site	N	B	SE	*P* value^*^	RR	95 % CI
Residual disease, in overall		1.466	0.453	0.001	4.333	0.639–6.379
Micro-	14					
Macro-	96					
Residual disease in upper abdomen		1.099	0.408	0.007	3.000	0.526–3.724
Micro-	23					
Macro-	87					
Total	110					

^a^Logistic regression analysis. *N* number of cases, *B* beta coefficient, *SE* standard error, *RR* relative risk; *95* % *CI* 95 % confidence interval

Although surgeons have made more efforts regarding how to best describe optimal cytoreduction in ovarian cancer, we believe that it is still not clear as to what constitutes “maximal” cytoreduction, or how to improve the surgical approach among the different teams, institutions, and countries. Complete cytoreduction is not equally applicable between neoadjuvant chemotherapy followed by interval cytoreductive surgery and primary cytoreductive surgery. Neoadjuvant chemotherapy can eliminate carcinomatosis in the peritoneum if it is chemosensitive, but its effects differ in patients undergoing “maximal” cytoreduction and peritonectomy. In the present study, Team B resected patients with tumor masses measuring >1 cm but left the peritoneal carcinomatosis in the upper abdomen; this was considered suboptimal by Team A. Thus, different interpretations of the term “maximal” can result in different surgical outcomes.

**Fig. 4:**
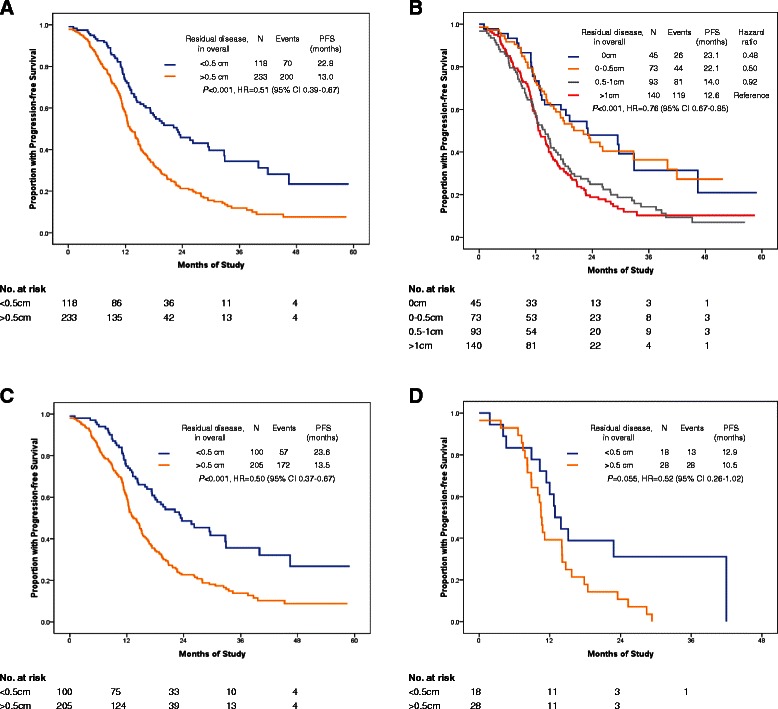
Survival analysis by residual disease in overall after primary cytoreductive surgery. **a**: PFS comparison of residual disease in overall among 0 cm, 0–0.5 cm, 0.5–1 cm, >1 cm; **b**: PFS comparison of residual disease in overall between <0.5 cm vs. >0.5 cm; **c**: PFS by residual disease in overall after primary cytoreductive surgery in patients with FIGO stage IIIC. **d** Progression-free survival by residual disease in overall after primary cytoreductive surgery in patients with FIGO stage IV

**Fig. 5:**
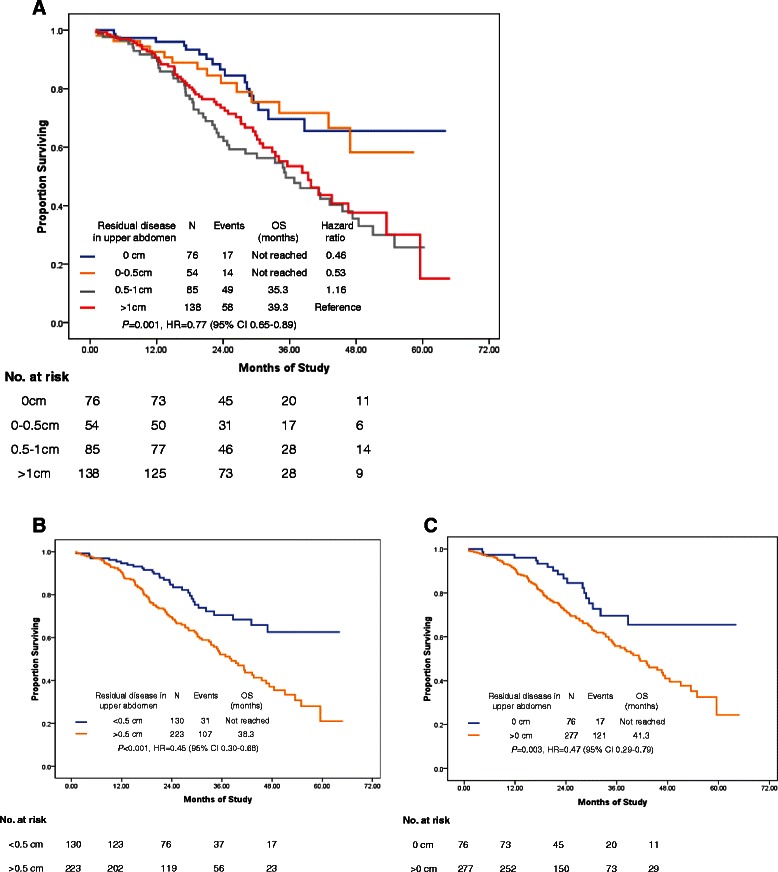
Overall survival by residual disease in upper abdomen after primary cytoreductive surgery. **a**: OS by residual disease in upper abdomen after primary cytoreductive surgery; **b**: OS by residual disease in upper abdomen with a comparison of cut-off point R0.5 cm; **c**: OS by residual disease in upper abdomen with a comparison of cut-off point R0 cm

**Fig. 6:**
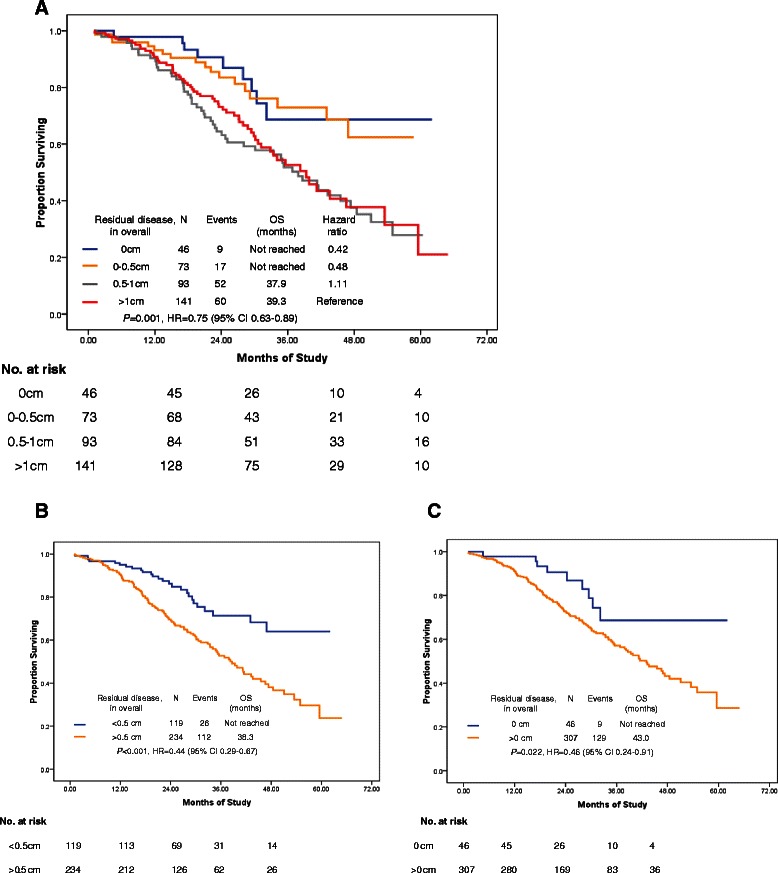
Overall survival by residual disease in overall after primary cytoreductive surgery. **a**: OS by residual disease in overall after primary cytoreductive surgery; **b**: OS by residual disease in overall with a comparison of cut-off point R0.5 cm; **c**: OS by residual disease in overall with a comparison of cut-off point R0 cm

In the radical surgery group, we considered the residual disease as ≤0.5 cm when peritoneal carcinomatosis was cytoreduced to microscopic residual by electronic knife, while not by en-block peritonectomy. We did not find a survival difference between lesions measuring 0.1–0.5 cm and 0 cm when residual disease was evaluated in either the upper abdomen alone or the whole abdomen (Figs. 3 and 4a). The common pattern of tumor involvement in the diaphragmatic and other upper regions of the abdominal peritoneum was carcinomatosis (339/353; 96 %); consequently, resection of all tumor nodes to a microscopic residual size in these patients was usually impossible. In our series, the cut-off point of 0.5 cm for residual disease is recommended for patients with bulky upper abdominal disease. This is because a biological complete cytoreduction is always rare when tumor cells have spread to the whole coelom (Figs. 4a, b; 5 and 6). However, a 41.1 % complete cytoreduction was achieved in the radical surgery group, so we should always keep in mind that complete gross cytoreductions are generally accepted, particularly in patients with less bulky UAD or less aggressive bowel carcinomatosis [3].

A longer operation time (≥4 h) did not improve the PFS, but shortened the OS, although not significantly (data not shown). Team A did 82 cases (73.2 %) radical surgery; and Team B only did 30 (26.8 %) radical surgery. There were 2 cases of relaparotomy for hemorrhage, 2 blood transfusion for hemorrhage, 1 intestinal fistula and 1 death in Team B, with no case in Team A. Therefore, EUAS performed by under-trained gynecologic surgeons is not recommended because it can result in more morbidity and mortality (Table 9).

Because of non-randomization, the weakness of our study was potential selection bias. To minimize the bias of the study, the basic characters of the two groups were analyzed in Table 1, and no significant difference was found between two groups in the median age, primary tumor, histology, tumor grade, ECOG performance status, ASA status, CA125 level, Neoadjuvant chemotherapy (NAC), ascites, and bowel mesenteric carcinomatosis (*p* > 0.05). More patients with stage IV disease were in radical surgery group, as stage IV disease required more radical surgery during the operation (18.8 % vs. 10.8 %, *p* = 0.045). It is still not clear whether or not patients with stage IV disease benefit from radical surgery (Fig. 4d). However, the results of the current study provide evidence for designing a randomized clinical trial.

## Conclusions

Extensive upper abdominal surgery lengthens the PFS and OS of ovarian cancer patients with bulky upper abdominal disease. Although these findings are based on short-term follow-up data, long-term follow-up is in progress. A well-designed randomized trial is needed to confirm the present results.

## Abbreviations

EUAS: Extensive upper abdominal procedures
UAD: Upper abdominal disease
EOC: Epithelial ovarian cancer
PFS: Progression-free survival
OS: Overall survival
HR: Hazard ratio
CI: Confidence interval
NAC: Neoadjuvant chemotherapy
FIGO: International Federation of Gynecology and Obstetrics
ECOG: Eastern Cooperative Oncology Group
ASA: American Society of Anesthesiologists
ICU: Intensive care unit
CT: Computed tomography
MRI: Magnetic resonance imaging
MSKCC: Memorial Sloan-Kettering Cancer Centre

## References

[1] Siegel R, Naishadham D, Jemal A (2013). Cancer statistics. CA Cancer J Clin.

[2] Chi DS, Eisenhauer EL, Lang J, Huh J, Haddad L, Abu-Rustum NR, Sonoda Y, Levine DA, Hensley M, Barakat RR (2006). What is the optimal goal of primary cytoreductive surgery for bulky stage IIIC epithelial ovarian carcinoma (EOC)?. Gynecol Oncol.

[3] du Bois A, Reuss A, Pujade-Lauraine E, Harter P, Ray-Coquard I, Pfisterer J (2009). Role of surgical outcome as prognostic factor in advanced epithelialovarian cancer: a combined exploratory analysis of 3 prospectively randomized phase 3 multicenter trials: by the Arbeitsgemeinschaft Gynaekologische Onkologie Studiengruppe Ovarialkarzinom (AGO-OVAR) and the Groupe d'Investigateurs Nationaux Pour les Etudes des Cancers de l'Ovaire (GINECO). Cancer.

[4] Rodriguez N, Miller A, Richard SD, Rungruang B, Hamilton CA, Bookman MA, Maxwell GL, Horowitz NS, Krivak TC (2013). Upper abdominal procedures in advanced stage ovarian or primary peritoneal carcinoma patients with minimal or no gross residual disease: an analysis of Gynecologic Oncology Group (GOG) 182. Gynecol Oncol.

[5] Barlin JN, Long KC, Tanner EJ, Gardner GJ, Leitao MM, Levine DA, Sonoda Y, Abu-Rustum NR, Barakat RR, Chi DS (2013). Optimal (≤1 cm) but visible residual disease: is extensive debulking warranted?. Gynecol Oncol.

[6] Hamilton CA, Miller A, Miller C, Krivak TC, Farley JH, Chernofsky MR, Stany MP, Rose GS, Markman M, Ozols RF, Armstrong DK, Maxwell GL (2011). The impact of disease distribution on survival in patients with stage III epithelial ovarian cancer cytoreduced to microscopic residual: a Gynecologic Oncology Group study. Gynecol Oncol.

[7] Zhang H, Yang T, Wu MC (2013). Surgical clinical trials--need for international collaboration. Lancet.

[8] Vergote I, Trope CG, Amant F, Kristensen GB, Ehlen T, Johnson N, Verheijen RH, van der Burg ME, Lacave AJ, Panici PB, Kenter GG, Casado A, Mendiola C, Coens C, Verleye L, Stuart GC, Pecorelli S, Reed NS, European Organization for Research and Treatment of Cancer-Gynaecological Cancer Group, NCIC Clinical Trials Group (2010). Neoadjuvant chemotherapy or primary surgery in stage IIIC or IV ovarian cancer. N Engl J Med.

[9] Rustin GJ, Vergote I, Eisenhauer E, Pujade-Lauraine E, Quinn M, Thigpen T, du Bois A, Kristensen G, Jakobsen A, Sagae S, Greven K, Parmar M, Friedlander M, Cervantes A, Vermorken J, Gynecological Cancer Intergroup (2011). Definitions for response and progression in Ovarian Cancer Clinical Trials Incorporating RECIST 1.1 and CA125 Agreed by the Gynecological Cancer Intergroup (GCIG). Int J Gynecol Cancer.

[10] Tang J, Liu DL, Shu S, Tian WJ, Liu Y, Zang RY (2013). Outcomes and patterns of secondary relapse in platinum-sensitive ovarian cancer: implications for tertiary cytoreductive surgery. Eur J Surg Oncol.

[11] Chi DS, Franklin CC, Levine DA, Akselrod F, Sabbatini P, Jarnagin WR, DeMatteo R, Poynor EA, Abu-Rustum NR, Barakat RR (2004). Improved optimal cytoreduction rates for stages IIIC and IV epithelial ovarian, fallopian tube, and primary peritoneal cancer: a change in surgical approach. Gynecol Oncol.

[12] Aletti GD, Dowdy SC, Podratz KC, Cliby WA (2006). Surgical treatment of diaphragm disease correlates with improved survival in optimally debulked advanced stage ovarian cancer. Gynecol Oncol.

[13] Chi DS, Eisenhauer EL, Zivanovic O, Sonoda Y, Abu-Rustum NR, Levine DA, Guile MW, Bristow RE, Aghajanian C, Barakat RR (2009). Improved progression-free and overall survival in advanced ovarian cancer as a result of a change in surgical paradigm. Gynecol Oncol.

[14] Eisenhauer EL, Abu-Rustum NR, Sonoda Y, Levine DA, Poynor EA, Aghajanian C, Jarnagin WR, DeMatteo RP, D'Angelica MI, Barakat RR, Chi DS (2006). The addition of extensive upper abdominal surgery to achieve optimal cytoreduction improves survival in patients with stages IIIC–IV epithelial ovarian cancer. Gynecol Oncol.

[15] Dowdy SC, Loewen RT, Aletti G, Feitoza SS, Cliby W (2008). Assessment of outcomes and morbidity following diaphragmatic peritonectomy for women with ovarian carcinoma. Gynecol Oncol.

